# Origin of music and embodied cognition

**DOI:** 10.3389/fpsyg.2015.00538

**Published:** 2015-04-28

**Authors:** Leonid Perlovsky

**Affiliations:** Department of Psychology, Northeastern UniversityBoston, MA, USA

**Keywords:** music origin, cognitive dissonance, musical emotions, embodied cognition, hierarchy of the mind, abstract thoughts, knowledge instinct

## Mystery of music

Music is a poorly understood ability. Its strong power over humans, its origin and cognitive function, have been a mystery for a long time. Aristotle (1995) listed the power of music among the great unsolved problems. Darwin wrote (1871) that musical ability “must be ranked amongst the most mysterious with which (man) is endowed.” Nature published a series of essays on music (Editorial, 2008). The authors of these essays agreed that “none… has yet been able to answer the fundamental question: why does music have such power over us?” (Ball, 2008).

In this article I advocate a hypothesis that music has a specific cognitive function to embody abstract thoughts. This embodiment proceeds through musical emotions, special types of emotions that we may experience when listening to music and that connect abstract thoughts and mental representations to instinctual drives. The embodiment of abstract thoughts through music is a unique contribution of this article.

## Aesthetic emotions and the knowledge instinct

According to a theory of drives and emotions developed by Grossberg and Levine (1987), instinctual drives are neural mechanisms similar to internal sensors in the mind-body. They measure vital bodily parameters and indicate to the organism their deviations from safe ranges. Emotional neural signals connect these instinctual indications to decision-making brain-mind regions (Grossberg and Levine, 1987; Grossberg, 1988). The emotions felt are associated with these neural signals. For example, a bodily sensor-like mechanism measures sugar level in the blood and indicates when it is below a safe range. This generates emotional neural signals, which are felt as hunger and drive decision-making mechanisms to look for food. Instincts and emotions are the mechanisms of embodiment.

This theory of drives and emotions has been extended to the knowledge instinct (KI) and aesthetic emotions (Perlovsky, 2001, 2006, 2007, 2014a,b; Perlovsky and Kozma, 2007; Mayorga and Perlovsky, 2008; Perlovsky et al., 2011). KI measures similarities between representations and top-down signals, or more generally between bottom-up and top-down signals. KI drives the development of mental representations in correspondence with experience throughout the hierarchy. Neural circuits involved in KI are discussed in Levine and Perlovsky (2008). Satisfaction or dissatisfaction of KI is indicated by aesthetic emotions. The existence of these special emotions related to knowledge were first discussed by Kant (1790), and have been experimentally demonstrated in Perlovsky et al. (2010). Aesthetic emotions embody knowledge.

## Mental hierarchy

Knowledge is stored in the brain's mental representations, which are organized in an approximate hierarchy from raw sensory percepts at the “bottom” to objects, to situations, to abstract thoughts, and to representations unifying life experience near the “top” of the mental hierarchy (Perlovsky, 2001, 2006; Perlovsky et al., 2011). Near the bottom of the hierarchy the knowledge is embodied in direct experience. “Higher up” representations of abstract knowledge cannot be embodied in direct experience, because abstract knowledge does not exist in the world “ready-made.” These representations are learned from personal experience under the guidance of language (Perlovsky, 2013b). Abstract knowledge exists in language, where it has been created during millennia of cultural evolution. Children learn knowledge at an early age through language; this is possible because real life experience is not needed. This difference between language and cognitive representations makes it possible for children to speak without “real” understanding.

Whereas language representations refer only to facts in language, cognitive representations refer to facts in the real world. Language representations become crisp and conscious in a child's mind by the age of 5 or 7. Cognitive representations, conversely, remain vague and less conscious (Perlovsky, 2013c). To appreciate this, one could imagine a familiar object with eyes closed; the imagination would be much more vague than the perception of the same object with opened eyes. Imaginations are neural projections from cognitive representations to the visual cortex (Grossberg, 1988); so vagueness of imaginations demonstrates vagueness of cognitive representations (Bar et al., 2006; Perlovsky, 2006, 2009). At higher levels in the mental hierarchy, abstract cognitive representations are even more vague and less conscious than language ability to clearly discuss these concepts. This is confirmed in brain imaging experiments (Binder et al., 2005): words for concrete objects stimulate cognitive brain areas in both hemispheres, whereas abstract words stimulate only language brain areas in the left hemisphere.

Therefore, abstract knowledge may remain as disembodied words, disconnected from the world and any emotions. To understand a concept, its cognitive representation must be developed and emotionally connected to the world, to instincts, and to life. Nonhuman animals have only embodied experience; instincts and emotions permeate their every move. But humans possess language, which makes abstract knowledge possible. The price for this ability is the differentiation of our psychic life. Language knowledge does not become automatically embodied and whole with cognition and emotion. Our psychic life is split. The human psyche is not as harmonious as the psyche of animals. Nietzsche (1876/1997) described our differentiated predicament as follows: “human is a dissonance.” Our psyche is not unified.

## Cognitive dissonance and emergence of music

At lower levels KI acts automatically: sensory-motor experiences are directly embodied. But at higher levels abstract knowledge is called abstract exactly because it does not exist pre-formed in the world, it is created through the interaction of the world and the mind. But cognitive dissonance (CD), a mechanism opposite to KI, might interfere at higher levels. CD is a discomfort caused by holding conflicting cognitions (Festinger, 1957; Cooper, 2007; Harmon-Jones et al., 2009). This discomfort is usually resolved by devaluing or discarding the conflicting cognition. This discarding often occurs below the level of consciousness; it is fast and momentary (Jarcho et al., 2011). It is also known that the majority of new knowledge originates through the differentiation of previous knowledge, which is the mechanism for several broad empirical laws: Zipf's law, the power law, Pareto law emerge when new entities (or usage) evolve from pre-existing ones (Simonton, 2000; Newman, 2005; Novak, 2010). Therefore, almost all knowledge contradicts other knowledge to some extent. According to CD theory, any knowledge should be discarded before its usefulness becomes established (Perlovsky, 2013a).

As language began emerging, every word brought new knowledge. However, CD should have interfered with this process. Before the usefulness of new knowledge could be established, it should have been discarded along with language (Perlovsky, 2013a). To overcome CD and enable the evolution of language and knowledge, a word should “grab attention” preconsciously. This is the purpose of prosody, or the melody of language sounds (Perlovsky, 2013b). This same mechanism is alive and well today. Over millions of years many musical and prosodial emotions have evolved and have been culturally inherited by everyone. Yet the diversity of culture is “too much” for some people, leading to new knowledge often being discarded in school and later. Recall that Einstein never received a Nobel Prize for Theory of Relativity. The emotions of language prosody embody the meanings of language and connect sounds to cognitive meanings in everyday conversations as well as in science (experimental evidence is discussed below). While language evolved toward more semantic and less emotional sounds, emotionality of voice, inherited from our animal past, evolved toward stronger emotions, to songs and music. And today song lyrics may affect us stronger than the same text without music.

## Experimental confirmations

Our hypothesis suggests that music evolved to connect abstract thoughts and KI, to embody abstract knowledge. This hypothesis has been confirmed experimentally. In Aronson and Carlsmith (1963) children devalued a toy if they could not play with it. The desire “to have” contradicts the inability “to attain”; this creates CD, which is resolved by discarding the contradiction. This experiment repeated many times (Cooper, 2007) was first described in Aesop's Fable 2500 years ago: the fox unable to attain the grape devalues a contradictory cognition by stating: “the grape is sour.”

Does music help in overcoming CD? Masataka and Perlovsky (2012a,b) have reproduced the above experiment with music in the background and observed that the toy is *not* devalued. Another experiment demonstrated that academic test performance may improve while listening to music. Perlovsky et al. (2013) demonstrated (1) that students allocate *less* time to more difficult and stressful tests (as expected from CD theory), and (2) with music in the background students *can* tolerate stress, allocate *more* time to stressful tests, and improve grades. These experiments confirmed the hypothesis that music helps in overcoming CD. It is likely that music emerged and evolved for a fundamental cognitive function: music makes the accumulation of knowledge and human evolution possible.

## Musical emotions

For thousands of years philosophers and psychologists wondered about the origin of dissonances and consonances. Masataka and Perlovsky (2013) demonstrated that consonant music helps “everyday” decision-making in the presence of cognitive interfering evidence, whereas dissonant music increases interference effects. Is music limited to a few emotions, or does every musical phrase evoke a different shade of emotion? Researchers take opposite sides of this issue (Scherer, 2004; Cross and Morley, 2008; Juslin and Västfjäll, 2008; Zentner et al., 2008; Koelsch, 2011; Juslin, 2013). As reviewed in Perlovsky (2012a,b), this does not affect the main argument of this paper for embodiment of abstract concepts through music.

How can multiplicity of emotions be explained and justified from a cognitive and evolutionary standpoint, and why has this ability emerged? The proposed hypothesis relating music to CD suggests the following explanation. CD produces a variety of emotional discomforts, different emotions for every combination of knowledge—in other words, a huge number of emotions. Most of these emotions are barely noticed because they lie below the level of consciousness, and in these unconscious states they produce disincentives for knowledge. Music helps to overcome these emotional discomforts by developing a huge number of conscious musical emotions. The mind being conscious of the multiplicity of emotions can bring into consciousness emotions of CD, and thus be prepared to tolerate them. We enjoy even sad and difficult musical emotions for their positive effect of overcoming difficult CD. Possibly this explains the mysterious enjoyment of sad music: it helps us to overcome CD of life's unavoidable disappointments, including the ultimate one, the knowledge of our finiteness in the material world.

Life is full of rhythms: heartbeat, respiration, day and night, and multiple other rhythms. Rhythms in music remind us that we are alive and yet most animals do not react to rhythms. Melody, harmony, and other musical devices produce complex, uniquely human musical emotions—they are related to knowledge and therefore are aesthetic emotions (Perlovsky et al., 2010; Bonniot-Cabanac et al., 2012). They expand KI toward a differentiated instinct sensitive not only to unifying knowledge and the world, but also to unifying multiplicity of contradictions among various aspects of knowledge. While CD split our psyche into differentiated knowledge, KI and music unify our psyche. Musical emotions embody abstract knowledge and unify our mental life, language and body.

These are the reasons why music affects us so strongly. Music connects thinking and intuition to the world. Our spiritual life is embodied through music. Uniquely human refined musical emotions embody our abstract thoughts from the everyday to the most exalted experience. Our highest mental representations near the top of the mental hierarchy attempt to unify our entire life experience. We perceive them as the meaning of life; their cognitive representations are vague but their feelings are strong, we feel them as emotions of the beautiful (Perlovsky, 2010a,b). These representations cannot be matched to anything “objectively existing” in the world outside of our brain-mind. Their deep meanings have been created in cultural evolution. Every individual human being receives this cultural knowledge through language. However, this cultural wisdom is not received in an embodied form. It might remain as meaningless disembodied text in books. It is up to everyone's personal effort to create an embodied meaning of life from one's own life experience. Music helps us to embody the meaning of life. The beautiful and sublime, art and religious experience, emotions that embody the meaning of life, as well as the highest spiritual experiences are all embodied through music.

### Conflict of interest statement

The author declares that the research was conducted in the absence of any commercial or financial relationships that could be construed as a potential conflict of interest.
